# Prevalence of Periodontitis and Its Association with Reduced Pulmonary Function: Results from the Korean National Health and Nutrition Examination Survey

**DOI:** 10.3390/medicina55090581

**Published:** 2019-09-10

**Authors:** Euni Lee, Seok-Woo Lee

**Affiliations:** 1 College of Pharmacy and Research Institute of Pharmaceutical Sciences, Seoul National University, Seoul 03080, Korea; 2 Departments of Dental Education and Periodontology, School of Dentistry, Dental Science Research Institute, Chonnam National University, Gwangju 61184, Korea

**Keywords:** chronic obstructive pulmonary disease, periodontitis, association, epidemiology, emphysema, neutrophil, neutrophil extracellular trap, oxidative stress, periodontal diseases, protease/proteinase

## Abstract

*Background and Objectives*: The current study was performed to evaluate the prevalence of periodontitis and to examine the association between reduced pulmonary function and periodontitis using Sixth Korea National Health and Nutrition Examination Survey (KNHANES) in 2014. *Materials and Methods*: A cross-sectional evaluation was conducted to estimate the prevalence of periodontitis and to examine the association between periodontitis and reduced pulmonary function while adjusting for sociodemographic characteristics and current smoking status in survey participants between 40 and 79 years old. The presence of periodontitis was evaluated by community periodontal index defined by the World Health Organization, and the assessments of reduced pulmonary function data were made as “normal,” “restrictive impairment,” or “obstructive impairment.” *Results*: A total of 4004 survey participants representing 25.4 million Koreans were included in the study. Overall, 41.1% of the study population were determined to have periodontitis, and 22.1% had reduced pulmonary function; 7.9% and 14.2% had restrictive- and obstructive- pulmonary impairments, respectively. Age, male gender, and current smoking status were positive predictors for periodontitis. Insurance coverage by workplace and higher education were protective factors against periodontitis. The association between periodontitis and restrictive impairment (adjusted odds ratio (OR) = 1.059, 95% CI 0.729–1.540) or obstructive impairment (adjusted OR = 1.140, 95% CI 0.849–1.530) was not significant. *Conclusions*: For Koreans, 40–79 years old, age, smoking status, gender, education, and insurance coverage were significant predictors of periodontitis. The prevalence of periodontitis was not significantly associated with reduced pulmonary function. To better understand the relationship between periodontitis and reduced pulmonary function, well-designed and larger scale epidemiologic studies are needed.

## 1. Introduction

Chronic obstructive pulmonary disease (COPD) is a generic term used to describe chronic lung diseases, including emphysema, chronic bronchitis, small airways disease, and non-reversible asthma [1]. It affects around 200 million people worldwide and is a major cause of morbidity and mortality [2]. COPD is characterized by airflow limitation that is usually progressive and associated with an abnormal inflammatory response of the lung to noxious particles or gases. The main cause of COPD is smoking, and it is aggravated by exacerbations likely caused by bacterial and/or viral infections [3]. Inflammation is considered to contribute to the etiology of COPD, as smoking or other toxic particles induce macrophage-predominant inflammation, airspace enlargement, and oxidative stress, leading to releasing proteases and tissue destruction [3].

Periodontitis is a chronic inflammatory disease that can lead to tissue destruction as a result of the perturbation of the homeostasis between the subgingival microflora and the host defense in susceptible individuals [4,5]. Immunological and inflammatory responses by the host to dental plaque biofilm via host-parasite interaction are manifested by signs and symptoms of periodontitis [6]. The initiation and progression of periodontitis are also influenced by genetic and environmental risk factors [7]. According to a recent Korean national disease statistics, the prevalence of periodontitis was 26.4% among adults aged 19 and older in 2014 [8].

Recently, more pieces of evidence have shown that a poor oral and periodontal condition is a potential risk factor for systemic diseases, strongly indicating the two-way interrelationship between oral and general systemic health [9,10,11]. For example, epidemiological and biological data indicate potential links between chronic periodontitis and systemic diseases, such as cardiovascular disease, diabetes, respiratory disease, rheumatoid arthritis, and adverse pregnancy outcomes [12,13,14,15].

A link between periodontitis and COPD has been proposed. Several studies have suggested that periodontitis is a co-factor for COPD [16]. It has also been observed that improved oral health may lower the frequency of COPD exacerbations in susceptible populations [17,18,19]. While a few studies reported associations between COPD and periodontitis, other studies showed no associations, and issues on diagnostic variability and data sources have also been raised [20,21]. Recent studies have suggested a strong association between COPD and periodontal disease. A systematic review study demonstrated that COPD patients suffer from worse periodontal condition [22]. A population-based study reported that the prevalence of periodontal disease was higher among COPD patients compared to non-COPD controls [23].

The purpose of the current study was to evaluate the prevalence of periodontitis and to examine the association between lung function test results, as indicators for COPD, and periodontitis using a national-level population data.

## 2. Materials and Methods

### 2.1. Data Source and Study Population

The study used a nationwide, population-based, cross-section survey known as the Korea National Health and Nutrition Examination Survey version 6 (KNHANES VI-1) data, specifically KNHANES 2014 data [8]. The survey data were collected by the Korea Centers for Disease Control and Prevention (KCDC) to examine the general health and nutritional status of Koreans since 1998 using a complex, stratified, and multistage probability-cluster sampling design representing non-institutionalized civilians in South Korea. Of the included three surveys of a health examination survey, a health interview survey and a nutritional survey were used and they included data obtained by direct physical examination, clinical and laboratory tests, and personal interviews [24].

Out of 7550 survey participants from the 2014 survey data, a total of 4004 participants, who were between 40 and 79 years old, were included in the analysis. This age group was determined as the study group because the pulmonary function test was collected only from these participants. Baseline characteristics, including sociodemographic characteristics (i.e., age, gender, education, marital status, and health insurance status), lifestyle factor on current smoking status, pulmonary function test assessments, and clinical condition of periodontitis, were collected. 

### 2.2. Definition of Pulmonary Function Test Assessment 

Pulmonary function test included measurements of forced vital capacity (FVC) and forced expiratory volume in one second (FEV_1_). Obstructive impairment was defined as FEV1/FVC < 0.70, and restrictive impairment was defined as FEV1/FVC ≥ 0.70 and FVC < 80%, while normal lung function was defined as FEV1/FVC ≥ 0.70 and FVC ≥ 80% [25,26]. A variable on the pulmonary function test assessment coded as “normal lung function”, “restrictive pulmonary impairment”, and “obstructive pulmonary impairment”, that was created and made publicly available by the KCDC, was used in the analysis (Table 1).

### 2.3. Definition of Periodontitis

The World Health Organization (WHO) community periodontal index (CPI) was used to assess the periodontal status [27]. Using a CPI probe satisfying the WHO guidelines, the presence of periodontitis was measured and defined as having a CPI greater than or equal to ‘‘code 3,” which indicates that at least one site had a periodontal pocket depth greater than 3.5 mm. CPI “code 4” had at least one site of pocket depth greater than 5.5 mm. The mouth was divided into sextants, and the index tooth numbers were 2, 3, 8, 14, 15, 18, 19, 24, 30, and 31. An approximately 25–35 g probing force was used for measuring a probing depth. Up to twenty dentist examiners participated in these measurements. The inter-examiner mean of Kappa value was 0.78, and the intra-examiner mean was 0.87.

### 2.4. Statistical Analysis

The analysis of the study focused on estimating the prevalence of recorded periodontitis diagnosis and evaluating its predictive factors. Using the unweighted sample data and the assigned sampling weight variable adjusted for the survey participants on pulmonary function test and complex sampling design, national-level estimates were calculated when they were aggregated to generate weighted frequencies. Descriptive statistics were used to calculate the frequencies of demographic characteristics and the prevalence of disease status. Univariable and multivariable logistic regression analyses were performed using weighted data. To assess the predictive factors for periodontitis diagnosis as the dependent variable (yes/no), a multivariable logistic regression model was constructed with pulmonary function test assessment as the independent variable with “normal lung function” as the reference group and determined the adjusted ORs with 95% confidence intervals (CIs) while adjusting confounders available from the data source and relevant to healthcare access, such as age, gender, current smoking status, level of education, marital status, and health insurance type, reflecting the Korean health insurance structure. 

All analyses in the study used weighted data to yield national-level frequencies using SPSS version 23.0 (SPSS Inc., Chicago, IL, USA). The significance level was set at *p* < 0.05. The study used publicly available national-level data without patient identifiers or linkable information. The study was approved by the Institutional Review Board from the Chonnam National University Dental Hospital, Gwangju, Korea (Approval number: CNUDH-EXP-2018-005, approved on 24 October 2018).

## 3. Results

From the KNHANES VI 2014 survey including 7550 study samples, a total of 4004 survey participants whose ages were between 40 and 79 years old representing 25.4 million Koreans were included in the study population. The mean (SD) age of the survey participants was 58.48 (SD 11.09). Demographic characteristics are described in Table 1. More female participants were recorded (male vs. female, 48% vs. 52%). Majority of the patients were married living with a spouse (85.7%) and covered by health insurance through their workplace (61.6%).

The prevalence of periodontitis was 41.1%. as determined by CPI. and 22.1% of the study population had reduced pulmonary function; 7.9% and 14.2% had restrictive- and obstructive- pulmonary impairments, respectively (Table 1).

Univariate and multivariate associations were evaluated by logistic regression analysis using weighted data. Age and current smoking status (adjusted odds ratio (OR) = 2.330, 95% CI 1.745–3.110) showed a statistically significant relationship with periodontitis (Table 2). Male participants were more likely to be diagnosed with periodontitis (adjusted OR = 2.045, 95% CI 1.618–2.585). Education level was also a strong predictor of periodontitis. Although significant associations were found from univariate analysis for higher level of education as a protective factor against periodontitis (unadjusted OR = 0.801, 95% CI 0.643–0.998 for middle school or less vs. high school graduate; unadjusted OR = 0.595, 95% CI 0.448–0.790 for middle school or less vs. college graduate or higher), college graduation or higher educational level was only a significant predictor in the adjusted model (adjusted OR = 0.598, 95% CI 0.423–0.846). Health insurance covered by workplace was also a protective factor against periodontitis (adjusted OR = 0.822, 95% CI 0.678–0.995) (Table 2). 

For the pulmonary function assessments, obstructive pulmonary impairment, but not restrictive pulmonary impairment, was a significant predictor of periodontitis from univariate analysis (unadjusted OR = 1.729, 95% CI 1.344–2.223). However, the association between periodontitis and restrictive pulmonary impairment (adjusted OR = 1.059, 95% CI 0.729–1.540) or obstructive pulmonary impairment (adjusted OR = 1.140, 95% CI 0.849–1.530) did not reach a statistical significance.

## 4. Discussion

In the present study, it was found that the prevalence of periodontitis was determined to be 41.1% among Korean population aged 40–79, as compared to 25.3% that was observed among adults aged over 19 in our previous study using identical national survey data [28]. This finding corroborates the fact that age is one of the most significant risk factors for periodontitis, as shown in numerous epidemiological and clinical studies [6,7,29].

In this study using a nationally representative population, we could not find any statistically significant association between reduced lung function and the prevalence of chronic periodontitis. Although several studies in the literature evaluated the association between pulmonary diseases and periodontitis [22,23,30], it has not yet been determined how these two maladies affect each other. Evidence of a positive association between COPD and periodontitis comes from a meta-analysis of 14 observational studies [31], where a pooled association OR was reported as 2.08 (95% CI 1.48–2.91). Another nationwide population-based cohort study by Shen et al. [32] reported that the overall incidence of periodontitis was 1.19-fold greater in COPD patients, compared with non-COPD patients. However, they defined the case of COPD based only on a questionnaire without measuring any reduced lung functions. The association can, therefore, be explained in part that periodontitis and COPD share several common risk factors, including smoking, age, and health behavior, as these factors may be involved in the initiation and progression of both diseases [33]. There are also reports suggesting an unclear relationship between COPD and periodontitis due to inadequate study designs and significant limitations in recognizing common risk factors, such as smoking [20,21]. Bergstrom et al. [33] failed to find an association between COPD and periodontitis, as they found that smoking was the major risk factor for periodontal pocket depth (OR 24.2; 95% CI 2–286.8). One NHANES III survey found no relationship between the two diseases among former or non-smokers [34]. It is plausible that defining causality between these disorders may be premature at present. Therefore, more definitive studies designed to establish causality and evaluate treatment outcomes are required to better understand a precise relationship between COPD and periodontitis.

The strengths of our study include the source of the data from national representative samples and the quality of the data from a quantitative analysis of the impaired and/or reduced lung function. More specifically, “obstructive” lung function (FEV_1_/FVC <70%) was used as a criterion for COPD, as defined by the Global Initiative for Chronic Obstructive Lung Disease (GOLD) guidelines, while other epidemiological studies adopted the value less than 65% of FEV_1_/FVC for COPD [35]. Although variabilities of cutoff value for defining “obstructive” lung function from the published studies may hinder a direct comparison between our findings and the results from other studies in assessing concrete relationships between COPD and periodontitis, KNHANES data could serve as a valuable research tool as it includes measurements of the lung function collected and coordinated by the KCDC following the GOLD guidelines that can be comparable to other healthcare-related data. 

A link between periodontitis and respiratory diseases, including COPD, has been proposed, as they may share common pathological processes resulting from immunological and inflammatory activities. It was suggested that periodontal microbes might play a role in the pathogenesis of COPD [36], as dental plaque bacterial species were identified in tracheal aspirate samples. Although the evidence of an independent association between chronic periodontitis and chronic obstructive pulmonary disease grows stronger, there remains a lack of definitive studies designed to establish causality and treatment effects. There is a need for future research to be focused on answering these questions. It was also found that oral hygiene interventions or periodontal treatment led to an improved condition of asthma and COPD [37,38,39]. Uncontrolled periodontal infection, characterized by the growth of dental plaque biofilms in periodontal tissues, may act as a constant source of chronic inflammation that can reach to other parts of the body beyond the oral cavity. It is, therefore, crucial to reduce and/or eliminate dental plaque biofilms to decrease the inflammatory burden to the whole body. This can be achieved by strict oral hygiene measures and routine periodontal care by dental professionals. Patients with COPD and periodontitis may require stricter and periodic follow-up, preferably through multidisciplinary collaborations. 

This study has several limitations, primarily due to the nature of the KNHANES IV as a secondary dataset. First, the periodontitis was assessed by the CPI, i.e., a simpler method of assessing the needs for periodontal treatment in a community setting. The CPI is reported to overestimate or underestimate the prevalence of periodontitis because the use of representative teeth does not truly reflect the whole periodontal condition [40]. Besides, the CPI scheme does not measure clinical attachment loss, i.e., a measurement of genuine periodontal tissue loss, and thus can underestimate the extent and severity of periodontal destruction that has previously occurred in the past [41]. Another limitation is derived from the nature of the cross-sectional design that does not allow clinical determination of COPD severity, and that makes impossible to determine the causal relationship between periodontitis and COPD. Therefore, the study can only describe the presence and the level of the association rather than describing the risk of developing periodontitis.

Despite several pieces of evidence suggesting a link between COPD and periodontitis, the exact mechanisms involving the association have not been fully elucidated. Therefore, well-designed future prospective observational studies or large scale big data analyses are required to clarify the causality, biological mechanisms, and clinical relationships in the future. Also, more research is required to improve and simplify technologies to assess the COPD condition reflecting the inflammatory and destructive process in the lung or to indicate responsiveness to treatment.

## 5. Conclusions

The results of the present study revealed that age, smoking status, gender, education, and insurance coverage were significant predictors of periodontitis. The reduced pulmonary function, both obstructive- and restrictive-pulmonary impairments, was not a significant predictor for the prevalence of periodontitis. Well-designed epidemiologic studies are needed to better understand a potential link and further find a causal relationship between periodontitis and obstructive pulmonary impairment.

## Figures and Tables

**Table 1:** Descriptive characteristics of the study population from 2014 KNHANES.

Characteristics	N (%) *
Total population	25,407,219 (100.0)
Age	
40–49	8,514,426 (33.5)
50–59	7,949,223 (31.3)
60–69	5,384,433 (21.2)
70–79	3,559,137 (14.0)
Gender	
Female	13,208,012 (52.0)
Male	12,199,207 (48.0)
Education	
Middle school or less	8,930,595 (37.9)
High school graduate	8,234,346 (34.9)
College graduate or higher	6,422,220 (27.2)
Marital status	
Single	751,615 (3.0)
Married	21,740,710 (85.7)
Divorced, separated, or widowed	2,868,036 (11.3)
Health insurance	
Self-employed	9,000,430 (35.7)
Workplace	15,508,011 (61.6)
Other ^†^	668,318 (2.7)
Current smoking status	5,141,912 (21.3)
Pulmonary function assessment	
Normal	19,796,391 (77.9)
Restrictive impairment	2,008,743 (7.9)
Obstructive impairment	3,602,085 (14.2)
Periodontal status	
**Periodontitis**	9,357,475 (41.1)

* Weighted frequencies were tabulated using sampling weight variable accounting for the complex sampling design provided by the KCDC (Korea Centers for Disease Control and Prevention). As not all demographic characteristics had complete records, aggregated frequencies for education, marital status, insurance status, current smoking status, and periodontitis were lower than the total population. ^†^ Health insurance accounted for national health insurance. ‘Other’ group includes Medical Aid class 1 and 2, no health insurance, or unknown. KNHANES: Korea National Health and Nutrition Examination Survey.

**Table 2:** Predictive factors for periodontitis.

Population Characteristics	Unadjusted OR (95% CI) *	Adjusted OR (95% CI) *
Age		
40–49	1	1
50–59	1.623 (1.314–2.005) ^†^	1.684 (1.300–2.181) ^†^
60–69	1.623 (1.242–2.121) ^†^	1.708 (1.241–2.352) ^†^
70–79	1.750 (1.307–2.344) ^†^	1.809 (1.244–2.631) ^†^
Pulmonary function assessment		
Normal	1	1
Restrictive impairment	1.402 (0.995–1.977)	1.059 (0.729–1.540)
Obstructive impairment	1.729 (1.344–2.223) ^†^	1.140 (0.849–1.530)
Gender		
Female	1	1
Male	2.503 (2.051–3.055) ^†^	2.045 (1.618–2.585)^†^
Education		
Middle school or less	1	1
High school graduate	0.801 (0.643–0.998) ^†^	0.861 (0.670–1.107)
College graduate or higher	0.595 (0.448–0.790) ^†^	0.598 (0.423–0.846) ^†^
Marital status		
Single	1	1
Married	1.106 (0.606–2.019)	0.339 (0.686–2.617)
Divorced, separated, or widowed	1.119 (0.577–2.169)	1.323 (0.633–2.764)
Health insurance		
Self-employed (community)	1	1
Workplace (employment)	0.779 (0.652–0.931) ^†^	0.822 (0.678-0.995) ^†^
Other	0.926 (0.535–1.602)	0.978 (0.543–1.762)
Smoking status		
No	1	1
Yes	2.834 (2.202–3.647) ^†^	2.330 (1.745–3.110) ^†^

* Odds Ratios (ORs) of the likelihood of periodontitis is based on weighted data. Adjusted OR refers to OR adjusted by all covariates included in the table. ^†^ *p* < 0.05. ‘Other’ group includes Medical Aid class 1 and 2, no health insurance, or unknown.
